# Shifting online during COVID-19: A systematic review of teaching and learning strategies and their outcomes

**DOI:** 10.1186/s41239-022-00361-7

**Published:** 2022-11-09

**Authors:** Joyce Hwee Ling Koh, Ben Kei Daniel

**Affiliations:** Higher Education Development Centre, University of Otago, 65-75 Union Place West, PO Box 56, Dunedin, 9054 New Zealand

**Keywords:** Online learning, E-learning, Emergency response teaching, COVID-19, Online dexterity, Online pedagogy

## Abstract

This systematic literature review of 36 peer-reviewed empirical articles outlines eight strategies used by higher education lecturers and students to maintain educational continuity during the COVID-19 pandemic since January 2020. The findings show that students’ *online access* and *positive coping strategies* could not eradicate their infrastructure and home environment challenges. Lecturers’ *learning access equity strategies* made learning resources available asynchronously, but having access did not imply that students could effectively self-direct learning. Lecturers designed *classroom replication, online practical skills training, online assessment integrity, and student engagement strategies* to boost online learning quality, but students who used ineffective *online participation strategies* had poor engagement. These findings indicate that lecturers and students need to develop more dexterity for adapting and manoeuvring their online strategies across different online teaching and learning modalities. How these online competencies could be developed in higher education are discussed.

## Introduction

Higher education institutions have launched new programmes online for three decades, but their integration of online teaching and learning into on-campus programmes remained less cohesive (Kirkwood & Price, 2014). Since early 2020, educational institutions have been shifting online in response to the COVID-19 pandemic. Some consider this kind of emergency remote teaching a temporary online shift during a crisis, whereas online learning involves purposive design for online delivery (Hodges et al., 2020). Two years into the pandemic, fully online, blended or hybridised modalities are still being used in response to evolving COVID-19 health advisories (Jaschik, 2021). Even though standards for the pedagogical, social, administrative, and technical requirements of online learning have already been published before the pandemic (e.g. Bigatel et al., 2012; Goodyear et al., 2001), the online competencies of lecturers and students remain critical challenges for higher education institutions during the pandemic (Turnbull et al., 2021). Emerging systematic literature reviews about higher education online teaching and learning during the pandemic focus on the clinical aspects of health science programmes (see Dedeilia et al., 2020; Hao et al., 2022; Papa et al., 2022). Understanding the strategies used in other programmes and disciplines is critical for outlining higher education lecturers’ and students’ future online competency needs.

This study, therefore, presents a systematic literature review of the teaching and learning strategies that lecturers and students used to shift online in response to the pandemic and their consequent outcomes. The review was conducted through content analysis and thematic analysis of 36 peer-reviewed articles published from January 2020 to December 2021. It discusses how relevant online competencies for lecturers and students can be further developed in higher education.

## Methodology

A Systematic and Tripartite Approach (STA) (Daniel & Harland, 2017) guided the review process. STA draws from systematic review approaches such as the Cochrane Review Methods, widely used in application-based disciplines such as the health sciences (Chandler & Hopewell, 2013). It develops systematic reviews through description (providing a summary of the review), synthesis (logically categorising research reviewed based on related ideas, connections and rationales), and critique (providing evidence to support, discard or offer new ideas about the literature).

### Framing the review

The following research questions guided the review:

1. What strategies did higher education lecturers and students use when they shifted teaching and learning online in response to the pandemic?
2. What were the outcomes arising from these strategies?

### Search strategy

Peer-reviewed articles were identified from databases indexing leading educational journals—Educational Database (ProQuest), Education Research Complete (EBSCOhost), ERIC (ProQuest), Scopus, Web of Science (Core Collection), and ProQuest Central. The following search terms were used to locate articles with empirical evidence of lecturers’ and/or students’ shifting online strategies:

- (remote OR virtual OR emergency remote OR online OR digital OR eLearning) AND (teaching strateg* OR learning strateg* OR shifting online) AND (higher education OR tertiary OR university OR college) AND (covid*) AND (success OR challenge OR outcome OR effect OR case OR lesson or evidence OR reflection)

The following were the inclusion and exclusion criteria:

- Review period—From January 2020 to December 2021, following the first reported case of COVID-19 (WHO, 2020).
- Language—Only articles published in the English language were included.
- Type of article—In order maintain rigour in the findings, only peer-reviewed journal articles and conference proceedings were included, and non-refereed articles and conference proceedings were excluded. Peer-reviewed articles reporting empirical data from the lecturer and/or student perspectives were included. Editorials and literature reviews were examined to deepen conceptual understanding but excluded from the review.
- The article’s focus—Articles with adequate descriptions and evaluation of lecturers’ and students’ online teaching and learning strategies undertaken because of health advisories during the COVID-19 pandemic were included. K-12 studies, higher education studies with data gathered prior to January 2020, studies describing general online learning experiences that did not arise from COVID-19, studies describing the functionalities of online learning technologies, studies about tips and tricks for using online tools during COVID-19, studies about the public health impact of COVID-19, or studies purely describing online learning attitudes or successes and challenges during COVID-19 without corresponding descriptions of teaching and learning strategies and their outcomes were excluded.

**Fig. 1:**
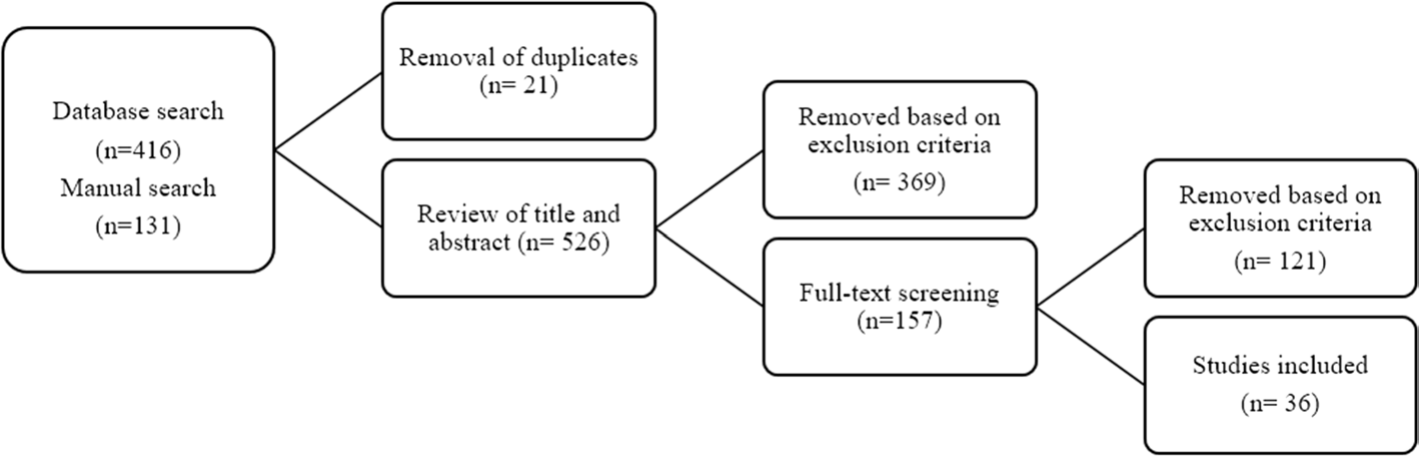
Article screening with the PRISMA process

A list of 547 articles published between January 2020 and December 2021 were extracted using keyword and manual search with a final list of 36 articles selected for review (see Fig. 1). The inclusion and exclusion criteria were applied to the PRISMA process (Moher et al., 2009). The articles and a summary of coding are found in Appendix.

### Data analysis

Content analysis (Weber, 1990) and thematic analysis (Braun & Clarke, 2006) were used to answer the research questions. Pertinent sections of each article outlining lecturers’ and/or students’ shifting online strategies were identified, read and re-read for data familiarisation. The first author used content analysis to generate eight teaching and learning strategies. These were verified through an inter-rater analysis where a random selection of eight articles was recoded by a second-rater (22.22% of total articles) and confirmed with adequate Cohen’s kappas (Teaching strategies: 0.88, Learning strategies: 0.78). Frequency counts were analysed to answer research question 1.

For the second research question, we first categorised the various shifting online outcomes described in each article and coded each outcome as “success”, “challenge”, or “mixed”. Successful outcomes include favourable descriptions of teaching, learning, or assessment experiences, minimal issues with technology/infrastructure, favourable test scores, or reasonable attendance/course completion rates, whereas challenging outcomes suggest otherwise. Mixed outcomes were not a success or challenge, for example, positive and negative experiences during learning, assessment or with learning infrastructure, or mixed learning outcomes such as positive test scores but lower ratings of professional confidence. Frequency distributions were used to compare the overall successes and challenges of shifting online (see Tables 1 and 2 of “Findings” section). Following this, the pertinent outcomes associated with each of the eight shifting online strategies were pinpointed through thematic analysis and critical relationships were visualised as theme maps. These were continually reviewed for internal homogeneity and external heterogeneity (Patton, 1990). To ensure trustworthiness and reliability (Creswell, 1998), there was frequent debriefing between the authors to refine themes and theme maps, followed by critical peer review with another lecturer specialising in higher education educational technology practices. Throughout this process, an audit trail was maintained to document the evolution of themes. These processes completed the description and synthesis aspects of the systematic literature review prior to critique and discussion (Daniel & Harland, 2017).

## Findings

### Descriptive characteristics

Descriptive characteristics of the articles are summarised in Table 1.

**Table 1:** Descriptive characteristics

Characteristic	N	% of total articles (N = 36)	
Publication period			
August–December 2020	15	41.67%	
January–June 2021	13	36.11%	
July–December 2021	8	22.22%	
Total	36	100%	
Site of study			
United States of America	11	30.56%	
Asia	9	25.00%	
Australasia	4	11.11%	
Middle East	3	8.33%	
Europe	2	5.56%	
Latin America	2	5.56%	
Africa	1	2.78%	
India	1	2.78%	
Canada	1	2.78%	
Multiple geographical locations	2	5.56%	
Total	36	100%	
Discipline			
Natural sciences	10	27.78%	
Medical and health sciences	6	16.67%	
Engineering or computer science	4	11.11%	
Commerce	3	8.33%	
Arts and social sciences	2	5.56%	
Education	1	2.78%	
Multiple disciplines	7	19.44%	
Others	3	8.33%	
Total	36	100%	
Programme level			
Diploma	1	2.78%	
Degree	21	58.33%	
Postgraduate (Master’s and Ph.D.)	4	11.11%	
Multiple programme levels	10	27.78%	
Total	36	100%	
Study methodology			
Quasi experiment/correlational study	4	11.11%	
Descriptive study	32	88.89%	
• Quantitative (n = 15)			
• Qualitative methods (n = 8)			
• Mixed methods (n = 9)			
Total	36	100%	
Sample size			
Below 50	13	36.11%	
51–149	10	27.78%	
150 or more	13	36.11%	
Total	36	100%	
Focus of paper			
Teaching strategies only	19	52.80%	
Learning strategies only	3	8.30%	
Both teaching and learning strategies	14	38.90%	
Total	36	100%	
Use of theoretical framing for teaching strategies			
Yes	7	19.44%	
No	29	80.56%	
Total	36	100%	

Shifting online outcomes	Definition	N	% of total articles (N = 36)
Online learning experiences	Successes or challenges experienced with synchronous or asynchronous learning activities	29	80.56%
Technology and infrastructure experiences	Successes or challenges experienced with computing equipment or home environment	16	44.44%
Assessment experiences	Successes or challenges experienced with online assessment	11	30.56%
Learning outcomes	Test scores or assessments of confidence, and/or learning attitudes	9	25.00%
Attendance/completion	Course attendance/completion rates	10	27.78%
Teaching workload	Teacher perceptions of workload arising from shifting online	3	8.33%

Table 1 shows that articles about shifting online during the pandemic were published steadily between August 2020 and December 2021. About two-thirds of the articles were based on data from the United States of America, Asia, or Australasia, with close to 45% of the articles analysing shifting online strategies used in the disciplines of Natural Sciences and Medical and Health Sciences and around 60% focusing on degree programmes. While there was an exact representation of studies with sample sizes from below 50 to above 150, the majority were descriptive studies, with close to half based on quantitative data gathered through surveys. About half of the articles focused on teaching strategies, while around 40% also examined students' learning strategies. However, only about 20% of the articles had theoretical framing for their teaching strategies. Besides using self-developed theories, the authors also used established theories such as the Community of Inquiry Theory by Garrison et. al. (2010), the Interaction Framework for Distance Education by Moore (1989), self-regulated learning by Zimmerman (2002) and the 5E model of Bybee et. al. (2006). Different types of shifting online outcomes were reported in the articles. The majority documented the positive and negative experiences associated with synchronous or asynchronous online learning activities, online learning technology and infrastructure, or online assessment. A quarter of the articles reported data on student learning outcomes and attendance/completion rates, while a minority also described teaching workload effects. Table 2 shows other successes and challenges associated with shifting online. Of the articles that examined online learning experiences, over a quarter reported clear successes in terms of positive experiences while about half reported mixed experiences. Majority of the articles examining technology and infrastructure experiences or assessment experiences either reported challenging or mixed experiences. All the articles examining learning outcomes reported apparent successes but only half of those investigating attendance/completion rates found these to be acceptable. Only challenges were reported for teaching workload.

**Table 2:** Success and challenges of shifting online

	Online learning experiences		Technology and infrastructure experiences		Assessment experiences		Learning outcomes		Attendance/completion		Teaching workload	
	N	%	N	%	N	%	N	%	N	%	N	%
Success	8	27.59	2	12.50	2	18.18	9	100	5	50.00	0	–
Mixed	15	51.72	3	18.75	5	45.46	0	–	4	40.00	0	–
Challenge	6	20.69	11	68.75	4	36.36	0	–	1	10.00	3	100
Total	29	100	16	100	11	100	9	100	10	100	3	100

### Teaching strategies and outcomes

Lecturers used five teaching strategies to shift online during the pandemic (see Table 3).

**Table 3:** Teaching strategies

Teaching strategies	No. of articles	% of total articles (N = 36)
1. Online practical skills training	9	25.00
2. Online assessment integrity	18	50.00
3. Classroom replication	21	58.33
4. Learning access equity	23	63.89
5. Student engagement	25	69.44

#### Online practical skills training

Lecturers had to create *online practical skills training*. With limited access to clinical, field-based, or laboratory settings, lecturers taught only the conceptual aspects of practical skills through online guest lectures, live skill demonstration sessions, video recordings of field trips, conceptual application exercises, or by substituting skills practice with new theoretical topics (Chan et al., 2020; de Luca et al., 2021; Dietrich et al., 2020; Dodson & Blinn, 2021; Garcia-Alberti et al., 2021; Gomez et al., 2020; Xiao et al., 2020). Only in three studies about forest operations, ecology, and nursing was it possible to practice hand skills in alternative locations such as public parks and students’ homes (Dodson & Blinn, 2021; Gerhart et al., 2021; Palmer et al., 2021).

*Outcomes*: Online practical skills training had different effects on learning experiences, test scores, and attendance/completion rates. Students can attain expected test scores through conceptual learning of practical skills (Garcia-Alberti et al., 2021; Gomez et al., 2020; Xiao et al., 2020). However, not all students had positive learning experiences as some appreciated deeper conceptual learning, but others felt disconnected from peers, anxious about losing hand skills proficiency, and could not maintain class attendance (de Luca et al., 2021; Dietrich et al., 2020; Gomez et al., 2020). Positive learning experiences, reasonable course attendance/completion rates, and higher confidence in content mastery were more achievable when students had opportunities to practice hand skills in alternative locations (Gerhart et al., 2021).

#### Online assessment integrity

Lecturers had to devise strategies to maintain *online assessment integrity*, primarily through different ways of preventing cheating (see Reedy et al., 2021). Pass/Fail grading, reducing examination weightage through a higher emphasis on daily work and class participation, and asking students to make academic integrity declarations were some changes to examination policies (e.g. Ali et al., 2020; Dicks et al., 2020). Randomising and scrambling questions, administering different versions of examination papers, using proctoring software, open-book examinations, and replacing multiple choice with written questions were other ways of preventing cheating during online examinations (Hall et al., 2021; Jaap et al., 2021; Reedy et al., 2021).

*Outcomes*: There was concern that shifting to online assessment had detrimental effects on learning outcomes, but several studies reported otherwise (Garcia-Alberti et al., 2021; Gomez et al., 2020; Hall et al., 2021; Jaap et al., 2021; Lapitan et al., 2021). Nevertheless, there were mixed assessment experiences. When lecturers changed multiple-choice to written critical thinking questions, it made students perceive that examinations have become harder (Garcia-Alberti et al., 2021; Khan et al., 2022). Some students were anxious about encountering technical problems during online examinations, while others felt less nervous taking examinations at home (Jaap et al., 2021). Students also became less confident about the integrity of assessment processes when lecturers failed to set clear rules for open-book examinations (Reedy et al., 2021). While Pass/Fail grading alleviated students’ test performance anxiety, some lecturers felt that this lowered academic standards (Dicks et al., 2020; Khan et al., 2022). More emphasis on daily work alleviated student anxiety as examination weightage was reduced, but students also perceived a corresponding increase in course workload as they had more assignments to complete (e.g. Dietrich et al., 2020; Swanson et al., 2021).

#### Classroom replication

Lecturers used *classroom replication* strategies to foster regularity, primarily through substituting classroom sessions with video conferencing under pre-pandemic timetables (Palmer et al., 2021; Simon et al., 2020; Zhu et al., 2021). Lecturers also annotated their presentation materials and decorated their teaching locations with content-related backdrops to emulate the ‘chalk and talk’ of physical classrooms (e.g. Chan et al., 2020; Dietrich et al., 2020; Xiao et al., 2020).

*Outcomes*: Regular video conferencing classes helped students to maintain course attendance/completion rates (e.g. Ahmed & Opoku, 2021; Garcia-Alberti et al., 2021; Gerhart et al., 2021). Student engagement improved when lecturers annotated on Powerpoint™ or digital whiteboards during video conferencing (Hew et al., 2020). However, screen fatigue commonly affected concentration, and lecturers had challenges assessing social cues effectively, especially when students turned off their cameras (Khan et al., 2022; Lapitan et al., 2021; Marshalsey & Sclater, 2020). Lecturers tried to shorten class duration with asynchronous activities, only to find students failing to complete their assigned tasks (Grimmer et al., 2020).

#### Learning access equity

Lecturers implemented *learning access equity* strategies so that those without stable network connections or conducive home environments could continue studying (Abou-Khalil et al., 2021; Ahmed & Opoku, 2021; Dodson & Blinn, 2021; Garcia-Alberti et al., 2021; Grimmer et al., 2020; Kapasia et al., 2020; Khan et al., 2022; Marshalsey & Sclater, 2020; Pagoto et al., 2021; Swanson et al., 2021; Yeung & Yau, 2021). They equalised learning access by making lecture recordings available, using chat to communicate during live classes, and providing supplementary asynchronous activities (e.g. Gerhart et al., 2021; Grimmer et al., 2020). Some lecturers only delivered lessons asynchronously through pre-recorded lectures and online resources (e.g. de Luca et al., 2021; Dietrich et al., 2020). In developing countries, lecturers created access opportunities by sending learning materials through both learning management systems and WhatsApp™ (Kapasia et al., 2020).

*Outcomes*: Learning access strategies maintained some level of student equity through asynchronous learning but created challenging student learning experiences. There is evidence that students could achieve expected test scores through asynchronous learning (Garcia-Alberti et al., 2021) but maintaining learning consistency was a challenge, especially for freshmen (e.g. Grimmer et al., 2020; Khan et al., 2022). Some students found it hard to understand difficult concepts without in-person lectures but they also did not actively attend the live question-and-answer sessions organised by lecturers (Ali et al., 2020; Dietrich et al., 2020; Gomez et al., 2020). Poorly designed lecture recordings and unclear online learning instructions from lecturers compounded these problems (Gomez et al., 2020; Yeung & Yau, 2021).

#### Student engagement

Lecturers used two kinds of *student engagement* strategies, one of which was through active learning. Hew et. al. (2020) fostered active learning through 5E activities (Bybee et al., 2006) that encouraged students to Engage, Explore, Explain, Elaborate, and Evaluate. Lapitan et. al. (2021) implemented active learning through their DLPCA process, where students Discover, Learn and Practice outside of class with content resources and Collaborate in class before Assessment. Chan et. al. (2020) used their Theory of Change to support active learning through shared meaning-making. Other studies emphasised active learning but did not reference theoretical frameworks (e.g. Martinelli & Zaina, 2021). Many described how lecturers used interactive tools such as Nearpod™, and Padlet™, online polling, and breakout room discussions to encourage active learning (e.g. Ali et al., 2020; Gomez et al., 2020).

Another student engagement strategy was through regular communication and support, where lecturers sent emails, announcements, and reminders to keep students in pace with assignments (e.g. Abou-Khalil et al., 2021). Support was also provided through virtual office hours, social media contact after class hours and uploading feedback over shared drives (e.g. Khan et al., 2022; Xiao et al., 2020).

*Outcomes*: Among the student engagement strategies, success in test scores tends to be associated with the use of active learning (Garcia-Alberti et al., 2021; Gomez et al., 2020; Hew et al., 2020; Lapitan et al., 2021; Lau et al., 2020; Xiao et al., 2020). On the other hand, positive learning experiences were more often reported when lecturers emphasised care and empathy through their communication (e.g. Chan et al., 2020; Conklin & Dikkers, 2021). Students felt this more strongly when lecturers used humour, conversational and friendly tone, provided assurance, set clear expectations, exercised flexibility, engaged their feedback to improve online lessons, and responded swiftly to their questions (e.g. Chan et al., 2020; Swanson et al., 2021). These interactions fostered the social presence of Garrison et. al.’s (2010) Community of Inquiry Theory (Conklin & Dikkers, 2021). However, keeping up with multiple communication channels increased teaching workload, especially when support requests arrived through social media after work hours (Garcia-Alberti et al., 2021; Khan et al. 2022; Marshalsey & Sclater, 2020).

### Learning strategies and outcomes

Students used three learning strategies during the pandemic (see Table 4).

**Table 4:** Learning strategies

Learning strategies	No. of articles	% of total articles (N = 36)
Online access	9	25.00
Online participation	15	41.67
Positive coping	5	13.89

#### Online access

Students had to maintain *online access*, as institutional support for data and technology was rarely reported (Ahmed & Opoku, 2021; Laher et al., 2021). Students did so by switching to more reliable internet service providers, purchasing more data, borrowing computing equipment, or switching off webcams during class (Kapasia et al., 2020; Mahmud & German, 2021).

*Outcomes*: Unstable internet connections, noisy home environments, tight study spaces, and disruptions from family duties were challenges often reported in students’ learning environments (e.g. Castelli & Sarvary, 2021; Yeung & Yau, 2021). The power supply was unstable in developing countries and students also had limited financial resources to purchase data. To keep studying, these students relied on materials shared through WhatsApp™ groups or Google Drive™ and learnt using mobile phones even though their small screen sizes affected students’ learning quality (Kapasia et al., 2020).

#### Online participation

Students had to maintain *online participation* by redesigning study routines according to when lecturers posted lecture recordings, identifying personal productive hours, changing work locations at home to improve focus and concentration, and devising study strategies to use online resources effectively, such as through note-taking (e.g. Abou-Khalil et al., 2021; Mahmud & German, 2021; Marshalsey & Sclater, 2020). Students also adjusted their online communication style by taking the initiative to contact lecturers through email, discussion forums, or chat for support, and learning new etiquette for video conferencing (Abou-Khalil et al., 2021; Dietrich et al., 2020; Mahmud & German, 2021; Simon et al., 2020; Yeung & Yau, 2021). Students recognised the need for active online participation (Yeung & Yau, 2021) but most tended to switch off webcams and avoided speaking up during class (Ahmed & Opoku, 2021; Castelli & Sarvary, 2021; Dietrich et al., 2020; Khan et al., 2022; Lapitan et al., 2021; Marshalsey & Sclater, 2020; Munoz et al., 2021; Rajab & Soheib, 2021).

*Outcomes*: Mahmud and German (2021) found that students lack the confidence to plan their study strategies, seek help, and manage time. Students also lacked confidence and switched off webcams out of privacy concerns or because they felt self-conscious about their appearances and home environments (Marshalsey & Sclater, 2020; Rajab & Soheib, 2021). Too many turned off webcams and this became a group norm (Castelli & Sarvary, 2021). Classes eventually became dominated by more vocal students, making the quieter ones feel left out (Dietrich et al., 2020).

#### Positive coping

Students’ *positive coping* strategies included family support, rationalising their situation, focusing on their future, self-motivation, and making virtual social connections with classmates (Ando, 2021; Laher et al., 2021; Mahmud & German, 2021; Reedy et al., 2021; Simon et al., 2020).

*Outcomes*: Positive coping strategies helped students to improve learning experiences, maintain attendance/completion rates, and avoid academic integrity violations during online examinations (Ando, 2021; Reedy et al., 2021; Simon et al., 2020). However, these strategies cannot circumvent technology and infrastructure challenges (Mahmud & German, 2021), while the realities of economic, family, and health pressures during the pandemic threatened their educational continuity and caused some to manifest negative coping behaviours such as despondency and overeating (Laher et al., 2021).

## Discussion

### Higher education online competencies

This systematic review outlined eight teaching and learning strategies for shifting online during the pandemic. Online teaching competency frameworks published before the pandemic advocate active learning, social interaction, and prompt feedback as critical indicators of online teaching quality (e.g. Bigatel et al., 2012; Crews et al., 2015). The findings suggest that lecturers’ student engagement strategies aligned with these standards, but they also needed to adjust practical skills training, assessment, learning access channels, and classroom teaching strategies. Students’ online participation and positive coping strategies reflected how online learners could effectively manage routines, schedules and their sense of isolation (Roper, 2007). Since most students had no choice over online learning during the pandemic (Dodson & Blinn, 2021), those lacking personal motivation or adequate infrastructure had to develop online participation and online access strategies to cope with the situation.

The eight teaching and learning strategies effectively maintained test scores and attendance/completion rates, but many challenges surfaced during teaching, learning, and assessment. Turnbull et. al. (2021) attribute lecturers’ and students’ pandemic challenges to online competency gaps, particularly in digital literacy or competencies for accessing information, analysing data, and communicating with technology (Blayone et al., 2018). However, the study findings show that digital literacy may not be enough for students to overcome infrastructure and home environment challenges in their learning environment. Lecturers can try helping students mitigate these challenges by providing asynchronous resource access through access equity strategies. Yet, students may not successfully learn asynchronously unless they can effectively self-direct learning. Lecturers may have pedagogical knowledge to create engaging active online learning experiences. How these strategies effectively counteract students’ inhibitions to turn on webcams and speak up during class remains challenging. Lectures may also have the skills to set up different online communication channels, but students may not actively engage if care and empathy are perceived to be lacking. Furthermore, lecturers’ online assessment strategies may not always balance academic integrity with test validity.

These findings show that online competencies are not just standardised technical or pedagogical skills (e.g. Goodyear et al., 2001) but “socially situated” (Alvarez et al., 2009, p. 322) abilities for manoeuvring strategies according to situation and context (Hatano & Inagaki, 1986). It encompasses “dexterity” or finesse with skill performance (Merriam-Webster, n.d.). The pandemic demands one to be “flexible and adaptable” (Ally, 2019, p. 312) amidst shifting national, institutional and learning contexts. Online dexterity is needed in several areas. Online learning during the pandemic is rarely unimodal. Establishing the appropriate synchronous-asynchronous blend is a critical pedagogical decision for lecturers. They need dexterity across learning modalities to create the “right” blend in different student, content, and technological contexts (Baran et al., 2013; Martin et al., 2019). Lecturers also need domain-related dexterity to preserve authentic learning experiences while converting subject content online (Fayer, 2014). Especially when teaching skill-based content under different social distancing requirements, competencies to maintain learning authenticity through simulations, alternative locations, or equipment may be critical (e.g. Schirmel, 2021). Dexterity with online assessment is also essential. Besides preventing cheating, lecturers need to ensure that online assessments retain test validity, improve learning processes and are effective for performance evaluation (AERA, 2014; Sadler & Reimann, 2018). Another area is the dexterity to engage in online communication that appropriately manifests care and empathy (Baran et al., 2013). Since online teaching increases lecturers’ workload (Watermeyer et al., 2021), dexterity to balance student care and self-care without compromising learning quality is also crucial.

Access to conducive learning environments critically affects students’ online learning success (Kapasia et al., 2020). While some infrastructure challenges cannot be prevented, students should have the dexterity to mitigate their effects. For example, when disconnected from class because of bandwidth fluctuations, students should be able to find alternative ways of catching up with the lecturer rather than remaining passive and frustrated (Ezra et al., 2021). Self-direction is critical during online learning because it is the ability to set learning goals, self-manage learning processes, self-monitor, self-motivate, and adjust learning strategies (Garrison, 1997). Students need the dexterity to manage self-direction processes across different courses, learning modalities, and learning schedules. Dexterity to create an active learning presence through using appropriate learning etiquette and optimising the affordances of text, audio, video, and shared documents during class is also essential. This can support students' cognitive, social, and emotional engagement across synchronous and asynchronous modalities, individually or in groups (Zilvinskis et al., 2017).

### Future directions

Online learning is highly diverse and increasingly dynamic, making it challenging to cover all published work for review. In this study, we have analysed pandemic-related teaching and learning strategies and their outcomes but recognise that a third of the studies were from the United States and close to half from natural or health science programmes. The findings cannot fully elucidate the strategies implemented in unrepresented countries or disciplines. Recognising these limitations, we propose the following as future directions for higher education:

#### Validate post-pandemic relevance of online teaching and learning strategies

The eight strategies can be validated through longitudinal empirical studies, theoretical analyses or meta-synthesis of literature to establish their relevance for post-pandemic teaching and learning. Studies outside the United States and the natural and health science disciplines are especially needed. This could address the paucity of theoretical framing in the articles reviewed, even with theories developed before the pandemic (e.g. Garrison et al., 2010; Moore, 1989; Zimmerman, 2002).

#### Demarcate post-pandemic online competencies

The plethora of descriptive studies in the articles reviewed is inadequate for understanding the online competencies driving lecturers’ pedagogical decision-making and students’ learning processes. In situ studies adopting qualitative methods such as grounded theory or phenomenology can better demarcate lecturers’ and students’ competencies for “why and under which conditions certain methods have to be used, or new methods have to be devised” (Bohle Carbonell et al., 2014, p. 15). A longitudinal comparison of these studies can provide a better understanding of relevant post-pandemic competencies.

#### Develop dexterity with respect to application of online competencies

Higher education institutions use technology workshops, mentoring, and instructional consultation to develop competencies in technology-enhanced learning (e.g. Baran, 2016). However, dexterity to manoeuvre contextual differences may be better fostered through exploration, discovery, and exposure to varied contexts of practice (Mylopoulos et al., 2018). Innovative ways of developing dexterity with respect to how online competencies can be applied and the efficacy of these methodologies are areas for further research.

## Conclusion

The COVID-19 pandemic has significantly increased the adoption and utilisation of online learning. While the present review findings suggest that the strategies lecturers and students employed to shift online during the pandemic have contributed to maintaining educational continuity and test scores but many outstanding issues remained unresolved. These include failure for students to gain an enhanced learning experience, problems encountered in designing and implementing robust assessment and online examinations, cases of academic misconduct, inequitable access to digital technologies, and increased faculty workload. Lecturers and institutions need to tackle these issues to fully leverage the opportunities afforded by online teaching and learning. Further, our findings revealed that the level of online dexterity for both students and teachers need to be enhanced. Therefore, higher education institutions must understand and develop online dexterity institutional frameworks to ensure that pedagogical innovation through online learning can be continually sustained, both during the pandemic and beyond.

## Data Availability

All data generated or analysed during this study are included in this published article.

## Appendix: Selected articles and coding

**Table:**

SN	Author and article information	Teaching strategies *ST—Online practical skills training* *AI—Online assessment integrity* *CR—Classroom replication* *AE—Learning access equity* *SE—Student engagement*					Learning strategies *OA—Online access* *OP—Online participation* *PC—Positive coping*			Outcomes *LE—Online learning experiences* *TIN—Technology and infrastructure experiences* *ASS—Assessment experiences* *LO—Learning outcomes* *AC—Attendance/Completion* *TW—Teaching workload* C—Challenge S—Success M—Mixed outcome					
		ST	AI	CR	AE	SE	OA	OP	PC	LE	TIN	ASS	LO	AC	TW
1	Abou-Khalil et. al. (2021) Site: Multiple Level: Multiple Subject: Multiple Methodology: Survey N: 300–349 Published: Jan-21 Theory: Moore’s interaction framework				√	√	√	√		M	C				
2	Ahmed and Opoku (2021) Site: Middle East Level: Multiple Subject: Engineering or Computer Science Methodology: Mixed methods N: 300–349 Published: Aug-21		√	√	√	√	√	√		M	M	M		S	
3	Ali et. al. (2020) Site: Australasia Level: Multiple Subject: Commerce Methodology: Qualitative N: ≤ 50 Published: Oct-20		√	√	√	√				M				M	
4	Ando (2021) Site: Asia Level: Postgrad Subject: Multiple Methodology: Qualitative N: ≤ 50 Published: Oct-20							√	√	M	C			S	
5	Castelli and Sarvary (2021) Site: USA Level: Degree Subject: Natural Sciences Methodology: Survey—Student N: 250–299 Published: Nov-20					√	√	√		C					
6	Chan et. al. (2020) Site: USA Level: Degree Subject: Natural Sciences Methodology: Qualitative N ≤ 50 Published: Aug-20 Theory: Theory of Change (ToC)	√		√	√	√				M					
7	Conklin and Dikkers (2021) Site: USA Level: Multiple Subject: Multiple Methodology: Survey N: 400–449 Published: Mar-21 Theory: COI					√				S					
8	de Luca et. al. (2021) Site: Multiple Level: Degree Subject: Medical and Health Sciences Methodology: Survey—Teacher N: ≤ 50 Published: Jan-21	√	√	√	√	√				C				C	
9	Dicks et. al. (2020) Site: Others Level: Degree (1st yr) Subject: Natural Sciences Methodology: Mixed methods N: ≤ 50 Published: Aug-20		√								S	S			
10	Dietrich et. al. (2020) Site: Europe Level: Degree Subject: Natural Sciences Methodology: Survey N: 100–149 Published: Aug-20	√	√	√	√		√	√		S					
11	Dodson and Blinn (2021) Site: USA Level: Degree Subject: Natural Sciences Methodology: Survey N: 51–99 Published: Apr-21	√	√	√	√	√				M	C				
12	Garcia-Alberti et. al. (2021) Site: Latin America Level: Multiple Subject: Engineering or Computer Science Methodology: Mixed methods N: ≤ 50 Published: Feb-21	√	√	√	√	√				C	C	C	S	S	C
13	Gerhart et. al. (2021) Site: USA Level: Degree Subject: Natural Sciences Methodology: Mixed methods N: ≤ 50 Published: Dec-20	√		√	√					S			S	S	
14	Gomez et. al. (2020) Site: USA Level: Degree Subject: Medical and Health Sciences Methodology: Mixed methods N: ≤ 50 Published: Sep-20	√	√	√	√	√				M			S		
15	Grimmer et. al. (2020) Site: Australasia Level: Degree (1st yr) Subject: Others Methodology: Qualitative N: 300–349 Published: Nov-20			√	√	√				M	C			M	
16	Hall et. al. (2021) Site: USA Level: Postgrad Subject: Medical and Health Sciences Methodology: Quasi-experiment/correlational N: ≥ 450 Published: Sep-21		√										S		
17	Hew et. al. (2020) Site: Asia Level: Postgrad Subject: Education Methodology: Quasi-experiment/correlational N: 51–99 Published: Dec-20 Theory: 5E			√		√				S			S		
18	Jaap et. al. (2021) Site: Europe Level: Degree Subject: Medical and Health Sciences Methodology: Quasi-experiment/correlational N: 100–149 Published: Feb-21		√				√				S	M	S		
19	Kapasia et. al. (2020) Site: Others Level: Multiple Subject: Multiple Methodology: Survey N: 200–249 Published: Sep-20				√	√	√				C				
20	Khan et. al. (2022) Site: Middle East Level: Degree Subject: Natural Sciences Methodology: Qualitative N: 51–99 Published: Oct-21		√	√	√	√		√		M	M	C		M	C
21	Laher et. al. (2021) Site: Others Level: Degree Subject: Arts and Social Sciences Methodology: Survey N: 150–199 Published: Jun-21						√	√	√	C	C				
22	Lapitan et. al. (2021) Site: Asia Level: Degree Subject: Engineering or Computer Science Methodology: Survey N: 150–199 Published: Jan-21 Theory: Discover, Learn, Practice, Collaborate and Assess (DLPCA)		√	√	√	√		√		M		M	S		
23	Lau et. al. (2020) Site: Asia Level: Diploma Subject: Natural Sciences Methodology: Mixed methods N: 350–399 Published: Nov-20		√	√	√	√				S		C	S		
24	Mahmud and German (2021) Site: Asia Level: Degree Subject: Others Methodology: Mixed methods N: 300–349 Published: Jul-21 Theory: Self-regulated Learning						√	√	√	M	C			M	
25	Marshalsey and Sclater (2020) Site: Australasia Level: Degree Subject: Arts and Social Sciences Methodology: Qualitative N: 51–99 Published: Nov-20			√	√	√		√		M	C				C
26	Martinelli and Zaina (2021) Site: Latin America Level: Multiple Subject: Engineering or Computer Science Methodology: Mixed M: < 51 Published: Oct-21					√				S			S		
27	Munoz et. al. (2021) Site: Asia Level: Postgrad Subject: Commerce Methodology: Qualitative N: ≤ 50 Published: Apr-21 Theory: COI				√	√		√		M					
28	Pagoto et. al. (2021) Site: USA Level: Degree Subject: Multiple Methodology: Qualitative N: 51–99 Published: Aug-21		√	√	√	√				M	M				
29	Palmer et. al. (2021) Site: USA Level: Degree Subject: Medical and Health Sciences Methodology: Survey N: ≤ 50 Published: May-21	√		√								S			
30	Rajab and Soheib (2021) Site: Middle East Level: Multiple Subject: Medical and Health Sciences Methodology: Survey N: 300–349 Published: Feb-21							√		C					
31	Reedy et. al. (2021) Site: Australasia Level: Multiple Subject: Multiple Methodology: Survey N: ≥ 450 Published: Mar-21		√			√		√	√			M			
32	Simon et. al. (2020) Site: USA Level: Degree Subject: Natural Sciences Methodology: Survey N: ≤ 50 Published: Aug-20		√	√	√	√		√	√	S					
33	Swanson et. al. (2021) Site: USA Level: Degree Subject: Commerce Methodology: Survey N: 300–349 Published: Jul-21		√		√	√				M	C	M			
34	Xiao et. al. (2020) Site: Asia Level: Degree (1st yr) Subject: Natural Sciences Methodology: Mixed methods N: ≤ 50 Published: Aug-20	√	√	√	√	√							S	S	
35	Yeung and Yau (2021) Site: Asia Level: Multiple Subject: Multiple Methodology: Survey N: 100–149 Publication month: Jun-21				√		√	√		C	C	C			
36	Zhu et. al. (2021) Site: Asia Level: Degree Subject: Others Methodology: Quasi-experiment/correlational N: 200–249 Published: Aug-21			√	√	√				S					

## References

[CR1] Abou-Khalil V, Helou S, Khalife E, Chen MA, Majumdar R, Ogata H (2021). Emergency online learning in low-resource settings: Effective student engagement strategies. Education Sciences.

[CR2] AERA. (2014). Standards for educational and psychological testing. https://www.testingstandards.net/uploads/7/6/6/4/76643089/standards_2014edition.pdf

[CR3] Ahmed V, Opoku A (2021). Technology supported learning and pedagogy in times of crisis: The case of COVID-19 pandemic. Education and Information Technologies.

[CR4] Ali I, Narayan AK, Sharma U (2020). Adapting to COVID-19 disruptions: Student engagement in online learning of accounting. Accounting Research Journal.

[CR5] Ally M (2019). Competency profile of the digital and online teacher in future education. International Review of Research in Open and Distributed Learning.

[CR6] Alvarez I, Guasch T, Espasa A (2009). University teacher roles and competencies in online learning environments: A theoretical analysis of teaching and learning practices. European Journal of Teacher Education.

[CR7] Ando S (2021). University teaching and learning in a time of social distancing: A sociocultural perspective. Journal of Human Behavior in the Social Environment.

[CR8] Baran E (2016). Investigating faculty technology mentoring as a university-wide professional development model. Journal of Computing in Higher Education.

[CR9] Baran E, Correia A-P, Thompson A (2013). Tracing successful online teaching in higher education: Voices of exemplary online teachers. Teachers College Record.

[CR10] Bigatel PM, Ragan LC, Kennan S, May J, Redmond BF (2012). The identification of competencies for online teaching success. Journal of Asynchronous Learning Networks.

[CR11] Blayone TJB, Mykhailenko O, Kavtaradze M, Kokhan M, vanOostveen R, Barber W (2018). Profiling the digital readiness of higher education students for transformative online learning in the post-soviet nations of Georgia and Ukraine. International Journal of Educational Technology in Higher Education.

[CR12] Bohle Carbonell K, Stalmeijer RE, Könings KD, Segers M, van Merriënboer JJG (2014). How experts deal with novel situations: A review of adaptive expertise. Educational Research Review.

[CR13] Braun V, Clarke V (2006). Using thematic analysis in psychology. Qualitative Research in Psychology.

[CR14] Bybee R, Taylor J, Gardner A, Van Scotter P, Powell J, Westbrook A, Landes N (2006). The BSCS 5E instructional model: Origins, effectiveness, and applications. Colorado Springs: BSCS. International Journal of Man-Machine Studies.

[CR15] Castelli FR, Sarvary MA (2021). Why students do not turn on their video cameras during online classes and an equitable and inclusive plan to encourage them to do so. Ecology and Evolution.

[CR16] Chan BC, Baker JL, Bunagan MR, Ekanger LA, Gazley JL, Hunter RA, O'Connor AR, Triano RM (2020). Theory of change to practice: How experimentalist teaching enabled faculty to navigate the COVID-19 disruption. Journal of Chemical Education.

[CR17] Chandler J, Hopewell S (2013). Cochrane methods—Twenty years experience in developing systematic review methods. Systematic Reviews.

[CR18] Conklin S, Dikkers AG (2021). Instructor social presence and connectedness in a quick shift from face-to-face to online instruction. Online Learning.

[CR19] Creswell JW (1998). Qualitative inquiry and research design.

[CR20] Crews TB, Wilkinson K, Neill JK (2015). Principles for good practice in undergraduate education: Effective online course design to assist students’ success. Journal of Online Learning and Teaching.

[CR21] Daniel BK, Harland T (2017). Higher education research methodology: A step-by-step guide to the research process.

[CR22] de Luca K, McDonald M, Montgomery L, Sharp S, Young A, Vella S, Holmes MM, Aspinall S, Brousseau D, Burrell C, Byfield D, Dane D, Dewhurst P, Downie A, Engel R, Gleberzon B, Hollandsworth D, Nielsen AM, O'Connor L, Starmer D, Tunning M, Wanlass P, French SD (2021). COVID-19: How has a global pandemic changed manual therapy technique education in chiropractic programs around the world?. Chiropractic & Manual Therapies.

[CR23] Dedeilia A, Sotiropoulos MG, Hanrahan JG, Janga D, Dedeilias P, Sideris M (2020). Medical and surgical education challenges and innovations in the COVID-19 era: A systematic review. In Vivo.

[CR24] Dicks AP, Morra B, Quinlan KB (2020). Lessons learned from the COVID-19 crisis: Adjusting assessment approaches within introductory organic courses. Journal of Chemical Education.

[CR25] Dietrich N, Kentheswaran K, Ahmadi A, Teychene J, Bessiere Y, Alfenore S, Laborie S, Bastoul D, Loubiere K, Guigui C, Sperandio M, Barna L, Paul E, Cabassud C, Line A, Hebrard G (2020). Attempts, successes, and failures of distance learning in the time of COVID-19. Journal of Chemical Education.

[CR26] Dodson EM, Blinn CR (2021). Forest operations instructor and student perspectives on rapid transition from face-to-face to online learning in the US. International Journal of Forest Engineering.

[CR27] Ezra O, Cohen A, Bronshtein A, Gabbay H, Baruth O (2021). Equity factors during the COVID-19 pandemic: Difficulties in emergency remote teaching (ert) through online learning. Education and Information Technologies.

[CR28] Fayer L (2014). A multi-case study of student perceptions of online course design elements and success. International Journal for the Scholarship of Teaching & Learning.

[CR29] Garcia-Alberti M, Suarez F, Chiyon I, Feijoo JCM (2021). Challenges and experiences of online evaluation in courses of civil engineering during the lockdown learning due to the COVID-19 pandemic. Education Sciences.

[CR30] Garrison, D. R. (1997). Self-directed learning: Toward a comprehensive model. *Adult Education Quarterly*, *48*(1), 18–33. 10.1177/074171369704800103

[CR31] Garrison DR, Anderson T, Archer W (2010). The first decade of the community of inquiry framework: A retrospective. The Internet and Higher Education.

[CR32] Gerhart LM, Jadallah CC, Angulo SS, Ira GC (2021). Teaching an experiential field course via online participatory science projects: A COVID-19 case study of a UC California Naturalist course. Ecology and Evolution.

[CR33] Gomez E, Azadi J, Magid D (2020). Innovation born in isolation: Rapid transformation of an in-person medical student radiology elective to a remote learning experience during the COVID-19 pandemic. Academic Radiology.

[CR34] Goodyear P, Salmon G, Spector JM, Steeples C, Tickner S (2001). Competences for online teaching: A special report. Educational Technology Research and Development.

[CR35] Grimmer R, Pollard A, Rolls N (2020). COVID-19 induced change in higher education: Reflections on rapidly transitioning a first-year undergraduate academic literacies unit from face-to-face to online. Journal of Academic Language and Learning.

[CR36] Hall EAP, Spivey CP, Kendrex HP, Havrda DEP (2021). Effects of remote proctoring on composite examination performance among Doctor of pharmacy students. American Journal of Pharmaceutical Education.

[CR37] Hao X, Peng X, Ding X, Qin Y, Lv M, Li J, Li K (2022). Application of digital education in undergraduate nursing and medical interns during the COVID-19 pandemic: A systematic review. Nurse Education Today.

[CR38] Hatano G, Inagaki K, Stevenson HAH, Hakuta K (1986). Two courses of expertise. Child development and education in Japan.

[CR39] Hew KF, Jia C, Gonda DE, Bai S (2020). Transitioning to the “new normal” of learning in unpredictable times: Pedagogical practices and learning performance in fully online flipped classrooms. International Journal of Educational Technology in Higher Education.

[CR40] Hodges, C. B., Moore, S., Lockee, B. B., Trust, T., & Bond, M. A. (2020). *The difference between emergency remote teaching and online learning*. Educause Review. https://er.educause.edu/articles/2020/3/the-difference-between-emergency-remote-teaching-and-online-learning

[CR41] Jaap A, Dewar A, Duncan C, Fairhurst K, Hope D, Kluth D (2021). Effect of remote online exam delivery on student experience and performance in applied knowledge tests. BMC Medical Education.

[CR42] Jaschik, S. (2021, 16 August). *Delta variant raises questions as campuses start semester.* Inside Higher Ed. https://www.insidehighered.com/news/2021/08/16/delta-variant-raises-questions-colleges-about-reopening-plans

[CR43] Kapasia N, Paul P, Roy A, Saha J, Zaveri A, Mallick R, Barman B, Das P, Chouhan P (2020). Impact of lockdown on learning status of undergraduate and postgraduate students during COVID-19 pandemic in West Bengal, India. Children and Youth Services Review.

[CR44] Khan S, Kambris ME, Alfalahi H (2022). Perspectives of university students and faculty on remote education experiences during COVID-19—A qualitative study. Education and Information Technologies.

[CR45] Kirkwood A, Price L (2014). Technology-enhanced learning and teaching in higher education: What is ‘enhanced’ and how do we know? A critical literature review. Learning, Media and Technology.

[CR46] Laher S, Bain K, Bemath N, de Andrade V, Hassem T (2021). Undergraduate psychology student experiences during COVID-19: Challenges encountered and lessons learnt. South African Journal of Psychology.

[CR47] Lapitan LD, Tiangco CE, Sumalinog DAG, Sabarillo NS, Diaz JM (2021). An effective blended online teaching and learning strategy during the COVID-19 pandemic. Education for Chemical Engineers.

[CR48] Lau PN, Chua YT, Teow Y, Xue XJ (2020). Implementing alternative assessment strategies in chemistry amidst COVID-19: Tensions and reflections. Education Sciences.

[CR49] Mahmud YS, German E (2021). Online self-regulated learning strategies amid a global pandemic: Insights from Indonesian university students. Malaysian Journal of Learning and Instruction.

[CR50] Marshalsey L, Sclater M (2020). Together but apart: Creating and supporting online learning communities in an era of distributed studio education. International Journal of Art & Design Education.

[CR51] Martin F, Ritzhaupt A, Kumar S, Budhrani K (2019). Award-winning faculty online teaching practices: Course design, assessment and evaluation, and facilitation. The Internet and Higher Education.

[CR52] Martinelli, S. R., & Zaina, L. A. M. (2021). *Learning HCI from a virtual flipped classroom: Improving the students’ experience in times of COVID-19.* In ACM international conference proceeding series, virtual event. 10.1145/3472301.3484326

[CR53] Merriam-Webster. (n.d.). *Dexterity*. Retrieved December 8, 2021, from https://www.merriam-webster.com/dictionary/dexterity

[CR54] Moher D, Liberati A, Tetzlaff J, Altman DG, The PG (2009). Preferred reporting items for systematic reviews and meta-analyses: The PRISMA statement. PLOS Medicine.

[CR55] Moore MG (1989). Three types of interaction. The American Journal of Distance Education.

[CR56] Munoz KE, Wang MJ, Tham A (2021). Enhancing online learning environments using social presence: Evidence from hospitality online courses during COVID-19. Journal of Teaching in Travel & Tourism.

[CR57] Mylopoulos M, Kulasegaram K, Woods NN (2018). Developing the experts we need: Fostering adaptive expertise through education. Journal of Evaluation in Clinical Practice.

[CR58] Pagoto S, Lewis KA, Groshon L, Palmer L, Waring ME, Workman D, De Luna N, Brown NP (2021). STEM undergraduates’ perspectives of instructor and university responses to the COVID-19 pandemic in Spring 2020. PLoS ONE.

[CR59] Palmer TJ, Chisholm LJ, Rolf CG, Morris CR (2021). Deliberate practice and self-recorded demonstration of skill proficiency: One baccalaureate nursing school’s response to the COVID-19 pandemic. Nurse Education in Practice.

[CR60] Papa V, Varotto E, Galli M, Vaccarezza M, Galassi FM (2022). One year of anatomy teaching and learning in the outbreak: Has the Covid-19 pandemic marked the end of a century-old practice? A systematic review. Anatomical Sciences Education.

[CR61] Patton MQ (1990). Qualitative evaluation and research methods.

[CR62] Rajab MH, Soheib M (2021). Privacy concerns over the use of webcams in online medical education during the COVID-19 pandemic. Cureus.

[CR63] Reedy A, Pfitzner D, Rook L, Ellis L (2021). Responding to the COVID-19 emergency: Student and academic staff perceptions of academic integrity in the transition to online exams at three Australian universities. International Journal for Educational Integrity.

[CR64] Roper AR (2007). How students develop online learning skills. Educause Quarterly.

[CR65] Sadler I, Reimann N (2018). Variation in the development of teachers’ understandings of assessment and their assessment practices in higher education. Higher Education Research & Development.

[CR66] Schirmel J (2021). COVID-19 pandemic turns life-science students into “citizen scientists”: Data indicate multiple negative effects of urbanization on biota. Sustainability.

[CR67] Simon LE, Genova LE, Kloepper MLO, Kloepper KD (2020). Learning postdisruption: Lessons from students in a fully online nonmajors laboratory course. Journal of Chemical Education.

[CR68] Swanson SR, Davis JC, Gonzalez-Fuentes M, Robertson KR (2021). In these unprecedented times: A critical incidents technique examination of student perceptions’ of satisfying and dissatisfying learning experiences. Marketing Education Review.

[CR69] Turnbull D, Chugh R, Luck J (2021). Transitioning to e-learning during the COVID-19 pandemic: How have higher education institutions responded to the challenge?. Education and Information Technologies.

[CR70] Watermeyer R, Crick T, Knight C, Goodall J (2021). COVID-19 and digital disruption in UK universities: Afflictions and affordances of emergency online migration. Higher Education.

[CR71] Weber RP (1990). Basic content analysis.

[CR72] WHO. (2020). *Novel coronavirus (2019-nCoV) situation report -1*. World Health Organization. https://www.who.int/docs/default-source/coronaviruse/situation-reports/20200121-sitrep-1-2019-ncov.pdf?sfvrsn=20a99c10_4

[CR73] Xiao CL, Cai H, Su YJ, Shen LM (2020). Online teaching practices and strategies for inorganic chemistry using a combined platform based on DingTalk, Learning@ZJU, and WeChat. Journal of Chemical Education.

[CR74] Yeung MWL, Yau AHY (2021). A thematic analysis of higher education students’ perceptions of online learning in Hong Kong under COVID-19: Challenges, strategies and support. Education and Information Technologies.

[CR75] Zhu XQ, Shek DTL, Chan CHM (2021). Promoting service leadership qualities and well-being among university students through an online course during COVID-19 pandemic. International Journal of Environmental Research and Public Health.

[CR76] Zilvinskis J, Masseria AA, Pike GR (2017). Student engagement and student learning: Examining the convergent and discriminant validity of the revised national survey of student engagement. Research in Higher Education.

[CR77] Zimmerman BJ (2002). Becoming a self-regulated learner: An overview. Theory into Practice.

